# Supporting the evolution of infectious disease research

**DOI:** 10.1242/dmm.052112

**Published:** 2024-10-01

**Authors:** Kirsty Hooper

## Abstract

**Summary:** In anticipation of our Special Issue, ‘Infectious Disease: Evolution, Mechanism and Global Health’, we celebrate recent advances made in this field and the success of our Infectious Disease Journal Meeting.

Long-established infectious diseases − such as HIV, tuberculosis and malaria that annually kill millions of people; emerging or recently emerged infectious diseases, such as COVID-19; and the global health threat of antimicrobial-resistant pathogens − have reinforced scientific and public interest in infectious disease (Joshi, 2022; Mostowy, 2022). There is an urgent need to improve our understanding of the mechanisms of pathogenesis, and develop new strategies to prevent, diagnose and treat infection. DMM is ideally situated to support and encourage such ground-breaking basic and pre-clinical research in this area (Tobin, 2022). To highlight this research, we recently launched a dedicated subject collection that coincided with our 2023 Journal Meeting, ‘Host−pathogen interactions through an evolutionary lens', organised by Wendy Barclay (Imperial College London, London, UK), Sara Cherry (University of Pennsylvania, Philadelphia, PA, USA), DMM Editor David Tobin (Duke University, Durham, NC, USA) and Russell Vance (University of California, Berkeley, CA, USA). This meeting was initiated in response to the sophisticated use of evolutionary analyses to gain insight into the crucial interactions between pathogen and host, which lead to disease. It provided a unique opportunity to bring together leading experts in infectious disease, host−pathogen interactions and evolutionary biology to examine new insights into infectious diseases. By using this evolutionary lens the participants explored the history of human infectious disease, pathogen emergence, adapting virulence traits and diverse host responses, culminating in discussions around clinical consequences and therapeutic opportunities. Here, we reflect upon how these themes are demonstrated in recently published research and how we can continue to support advances in this field.

Closely monitoring pathogen emergence and evolution is key in our global preparation for future pandemics, alongside the development of tools that can be used to research emerging health threats. The COVID-19 pandemic catapulted scientific progress in virology, vaccine science and viral evolution, and the knowledge and tools generated will be invaluable for continued investigation of viral pathogens (Sanyal, 2023). For instance, advanced *in vivo* (Jeong et al., 2022) and *in vitro* (García-González et al., 2023) systems were developed to interrogate the varied pathogenesis of SARS-CoV-2 infection. Similar strategies can be adapted for investigating other pathogens that continue to evolve and evade modern medical interventions, such as *in vitro* systems to study cerebral malaria (Adams and Jensen, 2022) and automated microscopy workflows to study antibiotic-resistant *Shigella* in zebrafish (Lensen et al., 2023). By building this research toolkit, we are better prepared to circumvent future infectious disease threats.

One key aspect of monitoring pathogen emergence and evolution is evaluating virulence traits. This has been demonstrated by Link et al. (2024) as they mapped the effect of individual Zika virus (ZIKV) proteins on disease phenotypes in *Drosophila*, and identified virulence traits that might have contributed to the increased pathogenesis associated with the outbreak of Zika virus infection in 2015. The viral proteins investigated in this study had varying effects on disease phenotypes in *Drosophila*, mirroring the human pathology, as several factors can affect the severity of congenital ZIKV syndrome (reviewed by Marcelino et al., 2023). Interestingly, a recent study also tracked the cellular mechanisms underlying the neurodevelopmental impact of congenital ZIKV syndrome upon exposure of newborns to the virus, rather than exposure during gestation (Ferreira et al., 2024). Fungal pathogens also have innovative strategies of virulence, such as their use of different metals to fuel invasion and survival. However, host responses have expertly adapted to exploit this reliance on metals or harness metal toxicity to combat fungal infection (Alselami and Drummond, 2023).

“There have been so many technical advances, creative new approaches, and high-quality and expansive datasets in the past decade; and so the scientific community is really primed to understand infectious disease evolution and pathogenesis much more deeply. That's really exciting, and DMM would love to promote collaboration among people making new biological insights and doing fundamental research on host-microbe interactions with clinicians and others who are working to improve therapies, prevention, and public health.”

DMM Editor, David Tobin, Department of Molecular Genetics and Microbiology, Duke University School of Medicine, USA.

As pathogens develop more sophisticated virulence strategies, host responses adapt, creating a dynamic molecular arms race. A recent large-scale screen using *Drosophila* identified a multitude of genes associated with host survival after *Candida albicans* infection that encompassed a variety of cellular functions, the most prominent concerning genes involved in fucosylation (Glittenberg et al., 2022). *Drosophila* were also used to uncover that TOR signalling is activated locally and systemically to remodel host lipid metabolism following intestinal infection with *Pseudomonas entomophila* (Deshpande et al., 2022). This TOR activation acts independently of, yet alongside, the innate immune pathways to promote survival. This study highlights the diversity of local and systemic host responses required to fight infections. One integral innate immune response is pyroptosis, which has recently been reviewed by Brokatzky and Mostowy in the context of bacterial infection (Brokatzky and Mostowy, 2022).

By expanding our understanding of the molecular arms race between pathogens and hosts, we can make tangible steps in preventing, diagnosing and treating infectious disease. Malaria is a devastating disease that persists owing to the infectious parasite and mosquito vector evolving to become resistant to existing drugs and insecticides. A new class of antimalarial drugs has been shown to target an integral protein involved in sexual maturation of male parasites, which could be used to block malarial transmission via administration to patients, together with symptomatic therapies or by being delivered directly to mosquitoes (Yahiya et al., 2023). Drug-resistance is also increasingly prevalent in *Mycobacterium tuberculosis* strains (Ramakrishnan, 2023); so, Knudsen Dal et al. (2022) used a zebrafish embryo tuberculosis model to identify compounds comprising anti-TB activity and low toxicity *in vivo*. Alongside novel drugs and compounds to combat pathogen infection, there is increasing interest in targeting microbiota. McGrath et al. (2022) developed an innovative *in vitro* human intestinal model to demonstrate the protective effect of two strains of anaerobic commensal bacteria in the context of pathogenic *Escherichia coli* infection. Complementary mouse models have also been generated for the same purpose (Sivignon et al., 2022).

While we are making these leaps in infectious disease research, we must ensure that we do not neglect pathogens that have less impact in middle- and high-income countries. On one note, research into pathogens more prevalent in tropical climates, e.g. those causing malaria or Zika virus infection, must continue to be expanded, as well as lesser-known pathogens, such as the chikungunya virus (reviewed by Henderson Sousa et al., 2023). This will ensure that improvements in the clinical management of infectious disease have equitable and inclusive impact. On another note, the climate crisis is also expected to expand the geographical range of these tropical diseases, meaning that environmental and clinical strategies to control these diseases will have to be adapted.

Our 2023 Journal Meeting gave us much to consider in the field of infectious disease by demonstrating the strides that have been made in our understanding of host−pathogen interactions, as well as the translation of this knowledge to clinical outcomes and ongoing challenges. Alongside fostering community through our Journal Meetings and other charitable activities, DMM's core aim is to publish rigorous, high-quality research. In this vein, and following on from the success of the recent Journal Meeting, we are launching a Special Issue entitled ‘Infectious Disease: Evolution, Mechanism and Global Health‘ to amplify our commitment to this evolving and expansive field of research (Box 1 and Fig. 1).

**Box 1: DMM Special Issue on Infectious Disease: Evolution, Mechanism and Global Health**

DMM invites you to submit your latest research for our upcoming Special Issue, Infectious Disease: Evolution, Mechanism, and Global Health. The issue is being driven by DMM Editors Sumana Sanyal (University of Oxford, UK) and David Tobin (Duke University, USA), alongside Guest Editors Judi Allen (The University of Manchester, UK) and Russell Vance (University of California, Berkeley, USA). There is growing appreciation of the evolving interactions between pathogen and host that lead to clinical manifestations, tolerance or resolution of infectious disease. DMM welcomes Research and Resource articles using a range of model systems and approaches that expand our understanding of the molecular arms race that occurs between host and pathogen, to foster meaningful clinical progress in the prevention, diagnosis and treatment of infectious disease.

The issue will also include specially commissioned Reviews, Perspectives and Interviews from leaders in the field and will be widely promoted online and at key global conferences, guaranteeing maximum exposure for your work. It is scheduled for publication in summer 2025, although note that our continuous publication model means that DMM will publish your article as soon as it is ready; you will not have to wait for the rest of the issue to be complete. We look forward to receiving your breakthrough infectious disease research by Monday 20 January 2025.

**Figure:**
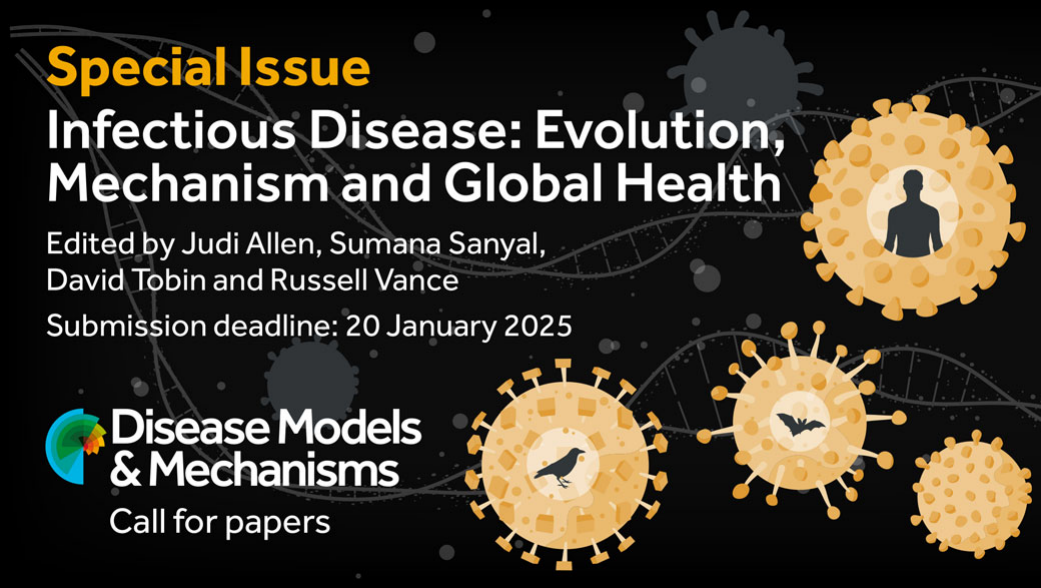
**Submit your research to our upcoming Special Issue, Infectious Disease: Evolution, Mechanism and Global Health, by Monday 20 January 2025.** This image is by neilsmithillustration.co.uk and published under the CC-BY 4.0 license for this article.
